# Plasmablast, Memory B Cell, CD4+ T Cell, and Circulating Follicular Helper T Cell Responses to a Non-Replicating Modified Vaccinia Ankara Vaccine

**DOI:** 10.3390/vaccines8010069

**Published:** 2020-02-06

**Authors:** Evan J. Anderson, Lilin Lai, Jens Wrammert, Sarah Kabbani, Yongxian Xu, Lalita Priyamvada, Heather Hill, Johannes B. Goll, Travis L. Jensen, Carol Kao, Inci Yildirim, Nadine Rouphael, Lisa Jackson, Mark J. Mulligan

**Affiliations:** 1 Departments of Pediatrics and Medicine, Emory University School of Medicine, Atlanta, GA 30322, USA; 2 Hope Clinic, Department of Medicine, Emory University School of Medicine, Decatur, GA 30030, USA; lilin.lai@nyulangone.org (L.L.); nfq8@cdc.gov (S.K.); y.xu@emory.edu (Y.X.); nroupha@emory.edu (N.R.); Mark.Mulligan@nyulangone.org (M.J.M.); 3 Department of Pediatrics, Emory University School of Medicine, Atlanta, GA 30322, USA; jwramme@emory.edu (J.W.); odk7@cdc.gov (L.P.); carol.kao@emory.edu (C.K.); inci.yildirim@emory.edu (I.Y.); 4 The Emmes Corporation, Rockville, MD 20850, USA; hhill@emmes.com (H.H.); jgoll@emmes.com (J.B.G.); bioinf@emmes.com (T.L.J.); 5 Kaiser Permanente, Seattle, WA 98101, USA; Lisa.A.Jackson@kp.org; 6 Langone Vaccine Center, New York University, New York, NY 10016, USA

**Keywords:** MVA, smallpox, follicular helper T cells (TFH), plasmablasts, vaccinia, antibody secreting cells

## Abstract

Background: Vaccinia is known to induce antibody and cellular responses. Plasmablast, circulating follicular helper T (cT_FH_) cells, cytokine-expressing CD4 T cells, and memory B cells were compared between subcutaneous (SC) and needle-free jet injection (JI) recipients of non-replicating modified vaccinia Ankara (MVA) vaccine. Methods: Vaccinia-naïve adults received MVA SC or by JI on Days 1 and 29. Vaccinia-specific antibodies were quantified by plaque reduction neutralization test (PRNT) and enzyme-linked immunosorbent assay. Plasmablast, cT_FH_, and cytokine-expressing CD4 T cells were assessed on Days 1, 8, 15, 29, 36, 43 (cT_FH_ and CD4+ only) and 57. Memory B cells were measured on Days 1 and 57. Results: Of the 36 enrolled subjects, only 22 received both vaccinations and had evaluable specimens after the second vaccine. Plasmablasts peaked one week after each vaccine. Day 15 plasmablasts correlated with peak PRNT titers. cT_FH_ peaked on Days 8 and 36 and correlated with Day 36 plasmablasts. CD4+ peaked at Day 29 and one-third produced ≥2 cytokines. Day 57 memory B cells ranged from 0.1% to 0.17% of IgG-secreting B cells. Conclusions: This study provides insights into the cellular responses to non-replicating MVA, currently used as a vector for a variety of novel vaccines.

## 1. Introduction

As a result of mass vaccination efforts, natural transmission of smallpox last occurred in 1977 and smallpox was declared eradicated by the World Health Organization in 1980 [1]. As vaccination with vaccinia halted due to smallpox eradication, the majority of the world’s population is now susceptible to this highly contagious and deadly disease, which is a potential agent of bioterrorism [2]. Although immunization with vaccinia virus resulted in excellent protection from smallpox, rare severe vaccine-related adverse reactions occurred [3,4]. A third-generation, non-replicating Modified Vaccinia Ankara (MVA) vaccine (previously known as IMVAMUNE®) was developed by Bavarian Nordic, Inc. (Morrisville, NC, USA) and is now licensed by the US Food and Drug Administration (FDA) (Jynneos™) and by the Europe Medicines Agency (IMVANEX). The vaccine virus has major deletions in its genome after repeated passage in chicken embryo fibroblast cells [5]. Thus, it is highly attenuated and does not replicate in most mammalian cells. A single dose of MVA resulted in seroconversion in >90% of individuals at 2 weeks, and two doses given at 0 and 4 weeks resulted in a robust, sustained humoral response [5,6]. The generation of long-lived plasma cells and memory B cells that maintain protective levels of antibodies for long periods of time is considered essential for most successful vaccines [7]. Prior studies with a replicating smallpox vaccine (Dryvax, Wyeth Pharmaceuticals, Inc., Philadelphia, PA) suggested that vaccinia-specific memory B cells can be detected 60 years after vaccination [8]. Although the development of vaccinia-specific antibodies correlates with immunity, data from B cell-deficient mice suggest that T cell responses are needed [9], and that these cellular responses may play a role in vaccine-related adverse reactions [8]. The interactions between cellular and humoral immune responses after initial MVA administration are poorly understood. 

Follicular helper T cells (T_FH_) are CD4+ T cells that express plasma membrane molecules (e.g., CXCR5, ICOS, PD-1), transcriptional regulators (e.g., BCL-6), and cytokines (e.g., IL-21, CD40L [CD154]) that shape B cell responses via lymphoid tissue germinal center reactions [10]. Circulating follicular helper T (cT_FH_) cells have been identified as human blood homologs of T_FH_ (BCL-6 negative) [10,11,12,13,14,15]. Bursts of plasmablasts (antibody-secreting cells) have been detected in the peripheral blood after vaccination and correlate with antibody response [16]. The correlation between the cT_FH_, plasmablasts, subsequent T cell memory, circulating memory B cells, and antibodies are not established for MVA. 

We previously reported a phase 2, open-label study that examined the safety and immunogenicity of MVA evaluating: (1) an accelerated MVA dosing schedule administered subcutaneously (SC) by needle versus the standard 4 week schedule; and (2) the use of a needle-free jet injector (Stratis™ Needle-free Injection System, PharmaJet, Golden, CO, USA) method of administration (JI) [17]. The purpose of this immunology substudy was to compare the magnitude, kinetics, functional qualities, and interplay of the primary and secondary immune responses (plasmablast, cT_FH_, T cells, memory B cells) induced by two doses of MVA administered on Days 1 and 29. In addition, we compared the plasmablast, memory B cell, and T_FH_ responses between subcutaneous needle-based administration and needle-free administration (Stratis™). 

## 2. Materials and Methods 

### 2.1. Study Design

This study was reviewed and approved by the Emory IRB. For the immunology substudy, participants were recruited from the phase 2 trial comparing two doses of MVA by the SC or JI routes [17]. Informed consent was obtained before any study procedures were conducted. Inclusion and exclusion criteria are listed on clinicaltrials.gov (NCT01827371). The MVA used in this study was lyophilized and was reconstituted with water for injection to a dose of 1 × 10^8^ TCID_50_ MVA per 0.5 mL [18]. Healthy, smallpox vaccine-naïve adults between 18 and 40 years of age were randomized to receive MVA on days 1 and 29 SC either by needle or by JI in the deltoid. The JI method is known to deliver the vaccine SC via a narrow, fluid stream. Subjects were enrolled between June 2013 and April 2015 (NCT01827371). 

### 2.2. Immunogenicity Assays

Bavarian Nordic performed vaccinia-specific individual plaque reduction neutralization tests (PRNT) and enzyme-linked immunosorbent assays (ELISAs) as previously described [17,19]. Two endpoints were analyzed for each subject: the first peak measurement endpoint (PEAK_1_) was defined as the highest PRNT (or ELISA) geometric mean titer (GMT) through the Day 29 visit, and the second peak measurement endpoint (PEAK_2_) was defined as the highest GMT from second vaccination through Day 57. Substudy participants agreed to provide additional blood sampling for the cellular assays on Days 1, 8, 15, 29, 36, 43, and 57.

Phenotypic plasmablast responses (antibody secreting cells (ASCs); as percentage of CD3^-^CD20^lo^CD19^+^CD38^hi^CD27^hi^ cells out of all CD19^+^CD3^-^ cells) were assessed with freshly isolated peripheral blood mononuclear cells (PBMCs) on Days 1, 8, 15, 29, 36 and 57 using flow cytometry [16]. Plasmablast enzyme-linked immunosorbent spot (ELISpot) assays were performed as previously described [7,16,20]. Memory B cell (MBC) responses (as percentage of vaccinia-responsive IgG-secreting cells out of total IgG-secreting B cells) were assessed at baseline and on day 57 using a 5 day in vitro polyclonal stimulation followed by detection and quantitation of MVA-responsive B cells by ELISpot [21]. 

The numbers and percentages of antigen-specific cT_FH_ cells (CD4+CXCR3+CXCR5+ ICOS+), or of antigen-specific CD4+ and CD4+CD154+(CD40L) T cells that produced certain cytokines (i.e., cells producing IFNγ, IL-2, IL-4, and/or IL-21 using individual and higher order combinations), were measured by intracellular staining (ICS) at days 1, 8, 15, 29, 36, 43 and 57. The antigen used for stimulation was 2 × 10^6^ pfu of Vaccinia WR kindly provided by Dr. Rama Amara (Emory Vaccine Center, Atlanta, GA). The numbers of total cytokine-producing T cells were calculated as the number of CTK+CD4+ T cells per 10^6^ total CD4+ T cells, or as the number of CTK+CD4+CD40L+ T-cells per 10^6^ total CD4+CD40L+ T cells (see Supplemental Methods). The degree of cytokine polyfunctionality was determined by calculating the average percentage for each cytokine function followed by normalization using the sum of the 4 averages to determine percentages.

### 2.3. Analysis Population

Analyses used the Modified According-To-Protocol (mATP) Analysis Population, which ensured exposure to vaccination and absence of major protocol deviations. Subjects with only one vaccination, or with a second vaccination that was out of window, were included for pre-second vaccination analyses but were excluded from post-second responses. When determining peak antibody responses, measurements collected outside the peak-specific windows (PEAK_1_: 13–31 days following first vaccination; PEAK_2_: 7–31 days following second vaccination) were excluded. For per-visit endpoint analyses, measurements collected outside the blood draw window were excluded.

### 2.4. Statistical Analysis

To capture all available immunogenicity data, results following dose 1 (through Day 29) are presented for all subjects who received a first vaccine dose. Data after dose 2 are presented for the subset of subjects who received two doses of vaccine in window and who had ≥2 blood samples collected between days 36 and 57. 

A two-sided Wilcoxon rank sum test was used to compare results between the SC and JI group. Peak responses following first vaccination (PEAK_1_) and post-second vaccination (PEAK_2_) were determined using the maximum results across the respective post-vaccination visits. PEAK results for PRNT and ELISA were summarized using GMTs and associated two-sided 95% confidence intervals (CI). Correlations between peak antibody, plasmablast, memory B cell, cT_FH_ cell, and cytokine-producing CD4+ T cells were determined using Spearman’s rank correlation. Results from subjects receiving MVA by SC and JI were pooled for the correlation analyses. Imputations were not performed to account for missing data. Significance was defined by *p* < 0.05. Results were obtained using SAS Version 9.3 and visualized using R Version 3.2.2. Enrollments into this immunology substudy were limited to a single site (Emory) due to the testing of ELISpot assays on live cells that were not previously frozen.

## 3. Results

### 3.1. Subject Study Details

Overall, we enrolled 36 subjects into this substudy: 14 men (nine in SC and five in JI) and 22 women (10 in SC and 12 in JI). The mean subject age was 29.1 ± 5.8 years (range: 19–40 years). As the purpose of this substudy was to learn more about cellular responses and to correlate these with humoral responses, we decided to present results based on the mATP population rather than the ITT population to account for vaccine administration adherence and other protocol deviations (see Methods section). Overall, the mATP analysis population included 33 evaluable subjects post-first vaccination and 22 subjects (13 SC and nine JI) post-second vaccination. For per-visit summaries, measurements collected outside the blood draw window were excluded from the mATP analyses. Results for the ITT population were similar (data not shown). Of the 12 (six in each study arm) who received only one vaccination, eight were due to a temporary study hold due to possible allergic reaction safety concerns [17]; two were due to adverse events; one was not eligible for the second vaccination; and one withdrew consent. Two subjects were not evaluable post-second vaccination. 

### 3.2. Antibody Responses 

PEAK_1_ PRNT was similar at Day 29 between subjects receiving MVA SC (GMT = 20 [95% CI: 9–45]) and by JI (GMT = 26 [95% CI: 13–49]). Following second vaccination, PEAK_2_ PRNT titer was lower in the SC group (GMT = 114 [95% CI: 59–222]) than in the JI group (GMT = 219 [95% CI: 122–395]) (Appendix Table A1). PEAK_1_ ELISA was lower by SC (GMT = 200 [95% CI: 118–338) than for JI (GMT = 378 [95% CI: 286–500]) administration, as was PEAK_2_ (SC GMT = 970 [95% CI: 636–1479]; JI GMT = 2401 [95% CI: 1286–4483]) (Appendix Table A2).

### 3.3. Plasmablasts (ASCs)

**Figure 1:**
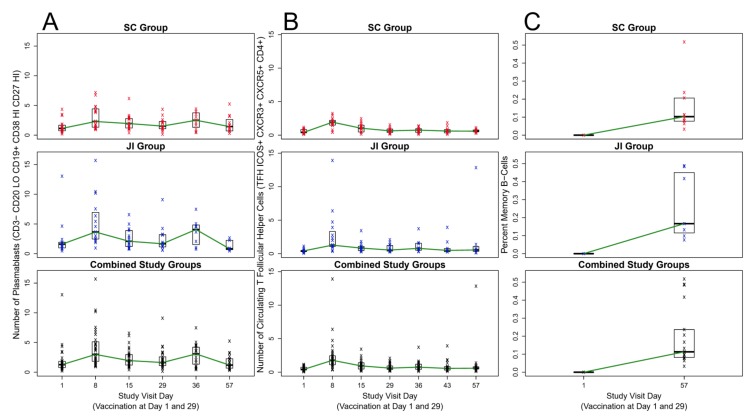
Plasmablast, Circulating T Follicular Helper (cT_FH_) Cell, and Memory B Cell Responses over Time After MVA Vaccination by Subcutaneous Injection and Jet Injection. Legend: JI = jet injection; SC: subcutaneous injection; *n* = 33 through Day 29; *n* = 22 from Day 36 to 57 (see methods for additional notes). (**A**) Number of Plasmablasts; (**B**) Number of Circulating T Follicular Helper (cT_FH_); (**C**) Circulating Memory B Cells Percentages.

Following first vaccination, similar peak percentages of plasmablasts were observed on Day 8 SC (1.51%) and JI (2.25%). Plasmablast percentages then declined through Day 29 (Figure 1A). Plasmablast percentages after the second vaccination on Day 36 increased for SC (1.49%) and JI (1.28%) compared to Day 29. 

### 3.4. cT_FH_ Cells

**Figure 2:**
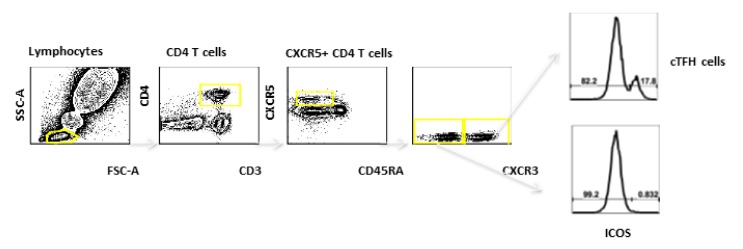
The Circulating T Follicular Helper (cT_FH_) Cell gating strategy for flow cytometry analyses of whole blood. Lymphocytes were identified by FSC-A/SSC-A after single cell gating. cT_FH_ Cells were defined by ICOS expression on CD3^+^CD4^+^CD45^-^CXCR5^+^CXCR3^+^ cells.

cT_FH_ Cells were defined by ICOS expression on CD3^+^CD4^+^CD45^-^CXCR5^+^CXCR3^+^ cells. (Figure 2). Prior to vaccination on Day 1, the median percentages of cT_FH_ cells were similar between the groups (3.04% in those receiving vaccine SC, 2.44% for those receiving vaccine by JI) (Figure 1B). Similar to plasmablasts responses, peaks in percentage cT_FH_ cells occurred at Day 8 for SC (median = 8.65%) and JI (median = 10.14%). A higher percentage of cT_FH_ cells was observed on Day 8 compared with Day 36 (SC median 4.90%, JI median 4.38%; Figure 1B). A peak timepoint for cT_FH_ cells was not observed after the second vaccination. 

### 3.5. MBCs 

At Day 1, the percentages of MVA-responsive IgG-secreting MBCs were undetectable (<0.001%) in both study groups. At Day 57, median percentages of MBCs increased to 0.103% for the SC and 0.167% for JI administration (Figure 1C).

### 3.6. ICS Assay

**Figure 3:**
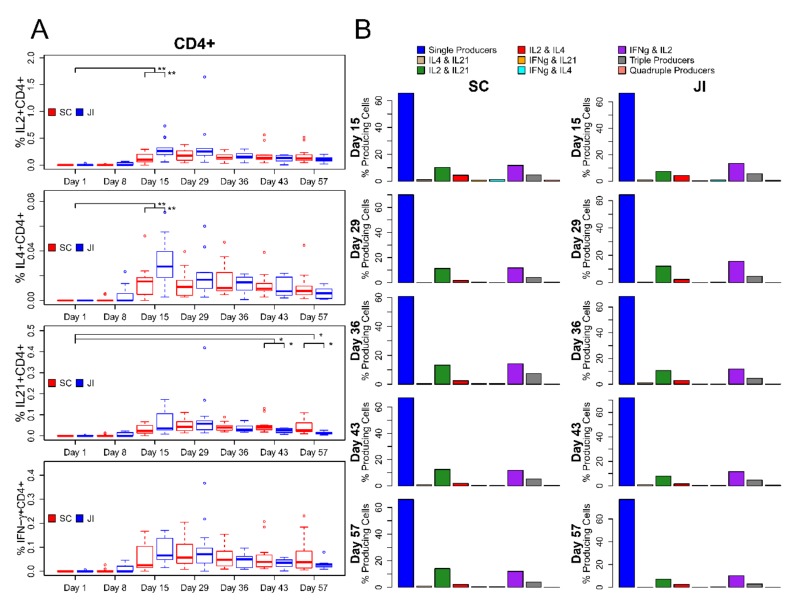
Changes in CD4+ T cell response by Treatment Arm and Polyfunctional Cytokine Response. (**A**). Boxplots of percent cytokine-expressing cells over time by treatment group. (**B**) Bar charts summarizing cytokine expression over time by treatment group. Vertical black lines indicate statistical significance based on Wilcoxon rank-sum test. *: *p* < 0.05, **: *p* < 0.01. Vertical lines that connect to Day 1 compare the difference in percent post- vs. pre-vaccination between treatment groups. Lines that connect treatment groups within time point compare percentages for that respective visit.

**Figure 4:**
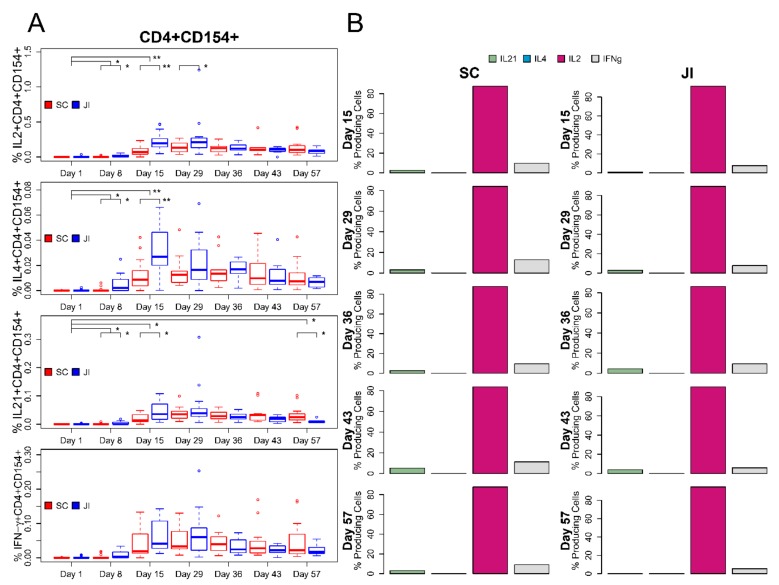
Changes in CD4+ CD154+ T cell response by Treatment Arm and Polyfunctional Cytokine Response (**A**). Boxplots of percent cytokine-expressing cells over time by treatment group. (**B**) Bar charts summarizing cytokine expression over time by treatment group. Vertical black lines indicate statistical significance based on Wilcoxon rank-sum test.*: *p* < 0.05, **: *p* < 0.01. Vertical lines that connect to Day 1 compare the difference in percent post- vs. pre-vaccination between treatment groups. Lines that connect treatment groups within time point compare percentages for that respective visit.

The median percentages of vaccinia-specific total cytokine-producing CD4^+^ T cells at Day 8 were similar to Day 1 (pre-vaccination) at <0.001% for both study arms. Median percentages of vaccinia-specific total cytokine-producing CD4^+^ cells showed initial responses at Day 15 with statistically significantly higher median percentages for those receiving vaccine by JI (median = 0.282%) compared with SC (median = 0.110%; Wilcoxon Rank-Sum test P-value = 0.004). These total cytokine-producing CD4+ cells remained elevated at Day 29 but declined by Day 43 in subjects who received a second vaccination. At baseline, the percentage of cytokine-producing CD4+CD154+(CD40L) T cells was very low (<0.001%). Subjects who received JI had a significantly higher percentage of CD4^+^CD154+ cells at Day 8 than those receiving SC (median of 0.013% versus <0.001%; Wilcoxon Rank-Sum test P-value = 0.038) and Day 15 (median of 0.327% versus 0.098%; Wilcoxon Rank-Sum test P-value = 0.007). The peak percentages of total antigen-specific cytokine-producing CD4+CD154+ cells occurred at Day 29 for both SC and JI (median of 0.226% versus 0.325%). The percentage of CD4+ cells that produced IFNγ, IL-2, IL-4, or IL-21 alone or in combination is demonstrated in Figure 3. The majority of antigen-specific cytokine-producing T cells (67.8%) produced a single cytokine, 27.1% produced two cytokines, 5.1% produced a polyfunctional CD4+ T-cell response with three (4.8%) or four cytokines (0.3%) when using the average of the medians across treatment groups and timepoints. The antigen-specific cytokine producing CD4+CD154+ cells produced by IL-2 most commonly followed by IFNγ, IL21, and IL4 is demonstrated in Figure 4. 

### 3.7. Correlations 

**Figure 5:**
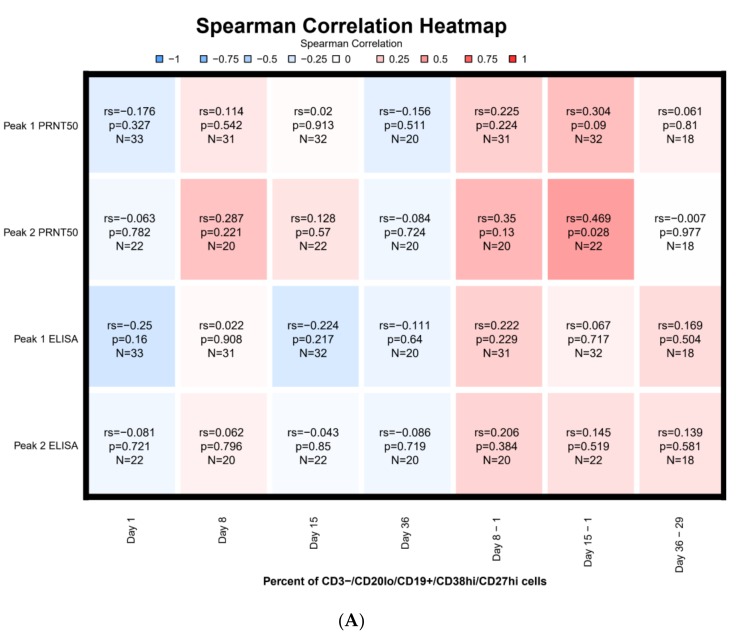

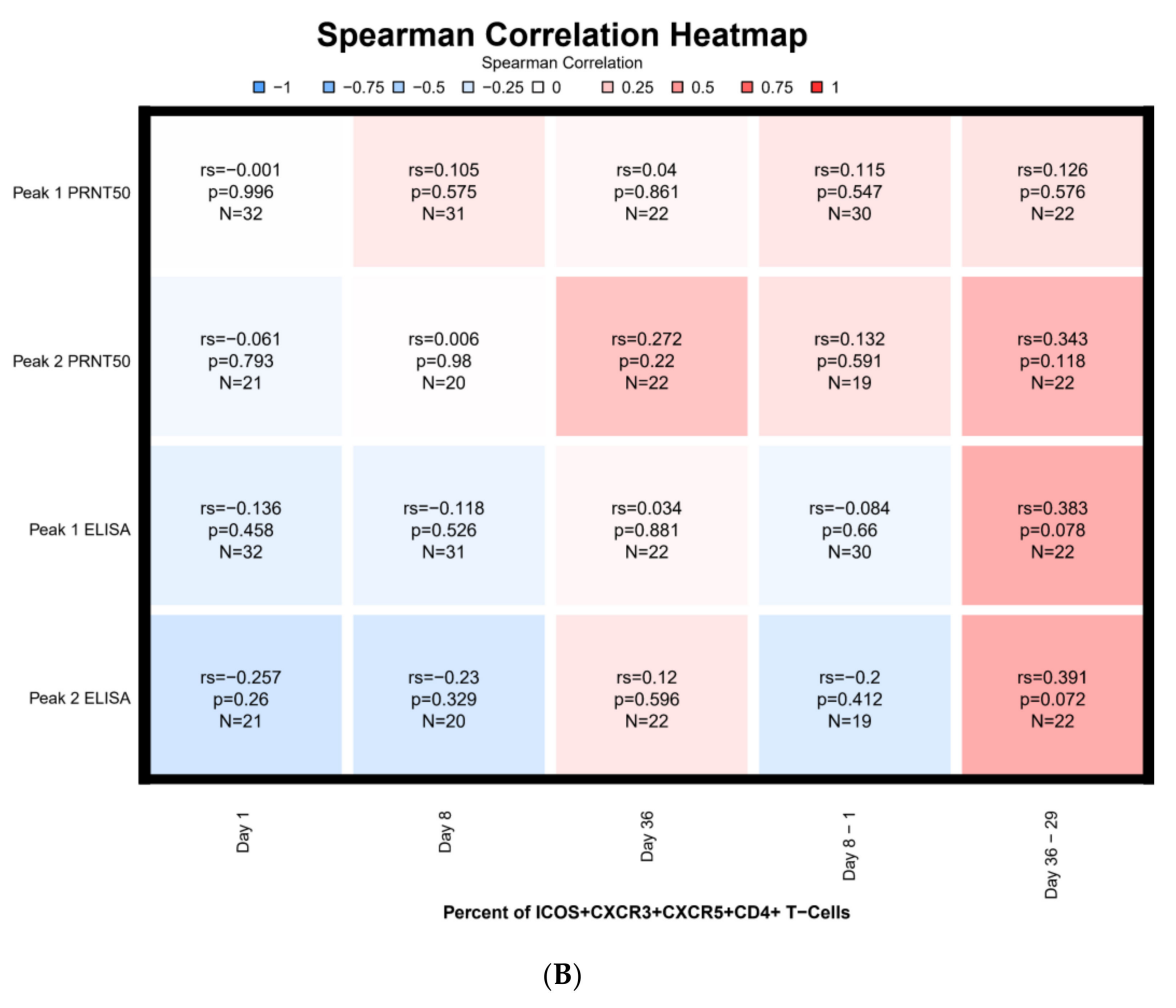
Serological Correlation with Plasmablast and Circulating T Follicular Helper (cT_FH_) Cells (**A**) Serologic Correlation with Plasmablast Responses. (**B**) Serologic Correlation with Circulating T Follicular Helper (cT_FH_) Cells. Legend: *n* = 33 through Day 29; *n* = 22 from Day 36 to 57. Each cell in the heatmap represents the Spearman correlation result between the percent plasmablast responses at a certain day and the respective antibody peak titer. Cell are color-coded by Spearman correlation. In red: positive correlations, in blue: negative correlations. Each cell includes the Spearman correlation, associated *p*-value, and number of samples for which both measured were available.

**Figure 6:**
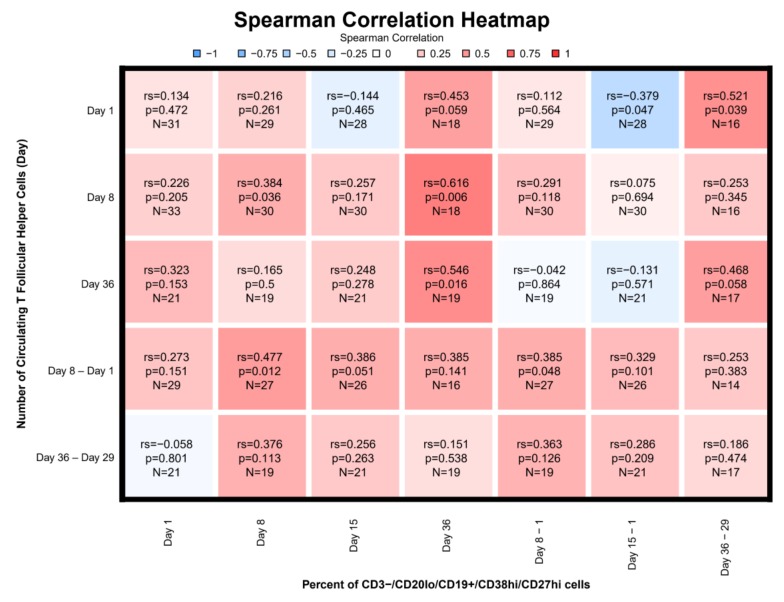
Plasmablast Correlation with Circulating T Follicular Helper (cT_FH_) Cells. Legend: *n* = 33 through Day 29; *n* = 22 from Day 36 to 57 (See caption of Figure 5A for further details).

The Day 15 differences in percentages of plasmablasts compared to pre-vaccination showed the strongest correlation with PEAK_1_ PRNT titer (r_s_ = 0.30) and PEAK_2_ PRNT titers (r_s_ = 0.47) (Figure 5A). The latter was statistically significant (*p* = 0.028). For subjects who received two doses of vaccine, the highest correlation for cT_FH_ cells and PRNT was observed for the percentage difference in cT_FH_ cells at Day 36 relative to Day 29 for PEAK_2_ ELISA (r_s_ = 0.39) followed by PEAK_1_ ELISA (r_s_ = 0.38), and PEAK_2_ PRNT (0.34) (Figure 5B). However, these did not reach statistical significance. Strongest statistically significant correlations were observed between Day 8 cT_FH_ numbers and percent of plasmablasts on Day 36 (r_s_ = 0.62), as well as between Day 36 cT_FH_ numbers and percent of plasmablasts on Day 36 (r_s_ = 0.55) (Figure 6). A weaker significant correlation was observed for both Day 8 cT_FH_ numbers as well as cT_FH_ Day 8-Day 1 numbers and Day 8 plasmablast percentages with r_s_ of 0.38 and 0.48, respectively. The difference in the percentage of plasmablasts between Day 15 and Day 1 inversely correlated with Day 1 cT_FH_ numbers (r_s_ = −0.38) (Figure 6). 

## 4. Discussion

Protection from smallpox by conventional vaccinia virus correlates with the development of a vesicular skin lesion at the site of vaccinia inoculation (a “take”), neutralizing antibody, and vaccinia virus-specific T cells, but the immunological correlates of protection against smallpox infection are not fully established as smallpox was eradicated before the development of modern techniques in immunology [22]. Prior studies suggest that both antibodies and cell-mediated immune responses are generated by vaccinia virus [22]. In prior clinical trials of MVA, the immunological response has been measured by development of vaccinia-specific IgG antibodies (ELISA), vaccinia-specific plaque reduction neutralization assay (PRNT), and vaccinia-specific interferon-gamma cytokine production by T cells by intracellular cytokine staining (ICS) [5]. Recent scientific advances made this study an excellent opportunity to better understand the cellular immunological responses and the interplay between plasmablasts, memory B cells, and cT_FH_ cells. 

T_FH_ shape the humoral immune response by producing cytokines such as CD154+(40L) that influence the IgG isotype produced by B cells [23]. A study of seasonal influenza vaccination [10] demonstrates that the cT_FH_ are antigen-specific, can induce memory B cells to differentiate into plasma cells, and correlate with antibody titers. In this study, the percent of cT_FH_ cells peaked at Day 8 and correlated with plasmablast responses at Day 36 and to a lesser extent at Day 8 (Figure 5). In addition, we found that cT_FH_ cell levels prior to vaccination inversely correlated with the change in the percent of plasmablasts observed at Day 15 (Figure 5). In this study, we did not find a statistically significant correlation between cT_FH_ and either peak antibody titers or MBCs at Day 57. This lack of correlation was surprising given the association noted between cT_FH_ and antibody titers and memory B cell differentiation into plasma cells that has been observed [10,11]. 

We observed a modest phenotypic plasmablast response after both the first and second vaccinations, similar in magnitude to that observed with influenza and yellow fever vaccines [16]. Although the peak plasmablast response occurred on Day 8, the increase in plasmablasts at Day 15 relative to pre-vaccination correlated with both peak PRNT_1_ and PRNT_2_ antibody responses. Plasmablast increases may occur earlier with recurrent dengue infection or after influenza revaccination [16,24], but may peak closer to Day 11–14 after yellow fever vaccination reflecting differences between primary and secondary immune responses [25]. Thus, one potential explanation for the relatively low percentage of plasmablasts at Day 8 is that we missed the peak (perhaps occurring between the Day 8 and 15 visits). Since plasmablast concentration differences at Day 15 relative to Day 1 correlated with peak PRNT titers, it is possible that improved plasmablast differentiation produced antibodies of greater specificity between Days 8 and 15, resulting in the better correlation with peak PRNT_1_ and PRNT_2_ antibody responses.

Vaccinia-specific CD4+ T cells increased across Days 8 and 15 with a peak at Day 29. CD4+ T cell numbers declined after the boost. It is possible that a longer interval between prime and boost would have allowed effector T cells, induced by the prime and peaking at day 29, to contract into memory cells that might have responded better to the boost. We further characterized the quality of the CD4+ T cell responses following vaccination by quantifying CD4+CD154+, and antigen-specific IFNɣ+, IL-21+, IL-2+, and IL-4+ producing CD4+ cells. These T cell responses increased from baseline until Day 29. Minimal increase occurred after the second dose of vaccine. The subjects who received JI vaccination had higher percentages of IFNɣ, IL-2, IL-4 and IL-21 producing CD4+ T cells on Day 15 relative to subjects who received needle-based SC vaccine. JI vaccines also had an earlier peak antigen-specific functional T cell response compared to needle-based vaccination. Variability within each group was high and the number of participants was low, so the significance, clinical relevance, and reasons for these differences in these data remain unknown. Although one study comparing JI versus needle and syringe delivery of BCG vaccine in adults and children did not find a difference in CD4 or CD8 T cell responses [26], several other studies suggest that a difference may be present. Using a recombinant adenovirus 5-vectored HIV DNA vaccine, higher responses were observed for Interferon gamma ELISPOT and envelope-specific antibodies [27]. In a Phase I study of two Zika DNA vaccines, needle-free vaccination resulted in higher GMTs compared to needle and syringe [28]. The relevance of this difference is unknown. 

The maintenance of protective levels of antibodies through the generation of long-lived plasma cells and MBCs is considered essential for most successful vaccines. Prior studies of MBC responses after replicating smallpox vaccine (Dryvax) showed that vaccinia-specific MBCs are detected at high numbers after vaccination and persist for as long as 60 years after vaccination, even in the absence of ongoing antigenic stimulation [7]. We observed a robust MBC response on Day 57 that was similar between groups. Unfortunately, cT_FH_, plasmablasts, and peak antibody titers did not predict subsequent MBCs levels at Day 57.

Limitations of these data include the relatively small sample size, particularly as one-third of subjects did not receive the second vaccine dose primarily due to the safety hold. This limited our ability to identify potential associations and correlations. The timing of phlebotomy here may have been too early to observe peak plasmablast circulation after the prime dose. Data from the primary study suggest that antibody responses are less robust with shorter intervals than 28 days between prime and boost [17], but it is possible that an even longer duration between prime and boost vaccination may provide further benefit in terms of cellular and antibody responses. Our last timepoint was at Day 57, so we cannot speculate about the induction of longer-term MBC and cellular responses by this nonreplicating MVA. Finally, immunological correlates of protection against smallpox are not known; but it is likely that multiple immune system components including cellular and humoral elements are involved in the protection against smallpox [22]. MVA recently resulted in similar ELISA and PRNT antibody responses as the established replicating vaccinia vaccine, ACAM2000, and it attenuated ACAM2000 cutaneous lesions after vaccination [6]. 

## 5. Conclusions

Our study expanded the knowledge base for cellular responses to the non-replicating MVA. Results from this study suggests that plasmablasts at Day 15 predict the peak antibody responses observed after the first and second dose of vaccine. CD4 T cell responses were observed. Additional data describing the interaction between cT_FH_, plasmablasts, T cells, and subsequent antibodies and establishment of memory B cell reservoirs would be of great immunological significance, particularly as MVA was recently licensed and is being used as a viral vector to administer a wide variety of proteins as a vaccination strategy (e.g., RSV, filoviruses, HIV). Exploration of these findings in future vaccine studies would be valuable. 

## Appendix A

### Phenotyping Assays

Each 200 μL of fresh whole blood were used for plasmablasts follicular helper CD4 T cells stains. Monoclonal antibodies for staining plasmablasts: from BD Biosciences: CD3 (SP34-2), CD19 (HIB19), CD38 (HIT2), from Biolegend: CD20 (2H7) and from eBioScience: CD27 (O323). Monoclonal antibodies for staining follicular helper CD4 T cells: from BD Biosciences: CD3 (UCHT1), CD4 (RPA-T4), CD8 (SK1), CD45RA (HI100), CXCR5 (RF8B2), CXCR3 (IC6/CXCR3) and CCR6 (11A9) From Biolegend: ICOS (C398.4A) and PD1 (EH12.2H7).

Data were collected on an LSRII (BD Biosciences) and analyzed using FlowJo software V9.7 (Tree Star).

### Intracellular Cytokine Staining

For measuring MVA-specific T cell responses, thawed PBMCs were rested overnight and then incubated for 6 h at 37 °C with ∼5 × 10^6^ PFU (multiplicity of infection of ∼2.5) of MVA in the presence of anti-CD28 and -CD49d (BD, # 555725 and #555501). After adding a cocktail containing brefeldin A and monensin (eBioscience, 004980-93), cultures were followed another 18 h. Negative-control samples were left unstimulated and positive-control samples were treated with Staphylococcal enterotoxin B (Sigma, #S4881) at a final concentration of 1 μg/mL PBMCs were then stained with Zombie cell viability dye (L423102, Biolegend). After fixation/permeabilization with Cytofix/Cytoperm, cells were stained with antibodies against: The cells were then stained with the following fluorescence conjugated antibodies: from BD Biosceinecs: CD3 (SP34-2), CD4 (L200) IL-2 (MQ1-17H12 and CD40L (TRAP1; from Biolegend IL-4 (MP4-25D2); from eBiosciences: IFN-gamma (4S. B3,) and IL-21(2A3-N2).After the cells for phenotyping and (ICS) cytokine staining experiments were washed, data were collected on an LSRII (BD Biosciences) and analyzed using FlowJo software V9.7 (Tree Star).

### Memory B Cells ELISpot Assays

Thawed PBMCs were cultured at 1 × 10^6^ cells per mL in R-10 supplemented with IL-2 and R488 (CTL-hBPOLYS-200, CTL) for 6 days. Cells were then placed in 96 well filter plates (Millipore, #MSHAN4B50) coated with MVA (2 × 10^6^ PFU/well). The plates incubated 6h at 37 °C. After twice washing in PBS and 4 times in PBS-Tween 20, plates were incubated for 2 h at room temperature with biotinylated anti-human IgG (Jackson Immunoresearch Laboratory, #709065098) at 1μg/mL diluted in PBST with 1% FBS. Plates were washed four times in PBST and incubated for 1h at room temperature with streptavidin-HRP (Vector Laboratories #A 2004) diluted 1:1000 in PBST with 1% FBS. Plates were washed three times each in PBST, then with PBS, followed with incubation with 3-Amino-9-ethylcarbazole (AEC) substrate kit (EMD Millipore Corporation, Substrate #152226, Buffer #152224) for 10 min until spot development. Plates were washed with water and allowed to dry; images were obtained using a CTL ELISpot plate reader. Total and virus-specific IgG-secreting MBCs were then quantified by ELISpot assay as described above.

## Appendix B

**Table A1:** PRNT Titers in JI Compared to SC.

	SC	JI
**PEAK_1_**		
*n*	18	15
Geometric Mean	20	26
Geometric 95% CI	9–45	13–49
**PEAK_2_**		
*n*	13	9
Geometric Mean	114	219
Geometric 95% CI	59–221	122–395

**Table A2:** ELISA Titers in JI Compared to SC.

	SC	JI
**PEAK_1_**		
*n*	18	15
Geometric Mean	200	378
Geometric 95% CI	118–338	286–500
**PEAK_2_**		
*n*	13	9
Geometric Mean	970	2401

PRNT: plaque reduction neutralization test (PRNT); ELISA: enzyme-linked immunosorbent assay. PEAK_1_: First peak measurement endpoint, which is defined as the highest PRNT (or ELISA) geometric mean titer (GMT) through the Day 29 visit; PEAK_2_: Second peak measurement endpoint, which is defined as the highest GMT from second vaccination through Day 57. JI: Jet Injection; SC: Subcutaneous injection.
